# Understanding High Achievement: The Case for Eminence

**DOI:** 10.3389/fpsyg.2019.01927

**Published:** 2019-08-27

**Authors:** Joseph Baker, Jörg Schorer, Srdjan Lemez, Nick Wattie

**Affiliations:** ^1^School of Kinesiology and Health Science, York University, Toronto, ON, Canada; ^2^Institute for Sport Science, Carl von Ossietzky University Oldenburg, Oldenburg, Germany; ^3^Department of Kinesiology and Health Promotion, California State Polytechnic University, Pomona, Pomona, CA, United States; ^4^Faculty of Health Sciences, Ontario Tech University, Oshawa, ON, Canada

**Keywords:** expertise, sport, development, athlete, training

## Abstract

The development of the field of sport expertise over the past 20 years has been remarkable, and our understanding of the varying factors affecting athlete development and motor skill acquisition has expanded considerably. Recently, there has been a push toward more sophisticated research designs to continue the advancement of our understanding of sport expertise. Even in a population of performers at the highest levels of performance and competition (e.g., participants in professional sports or those who compete at Olympic Games), there are those with obvious superiority compared to others in the cohort, such as those who win “most valuable player” awards or who are elected to the Hall of Fame. This paper builds a case that athletes who reach this level of achievement possess a more advanced level of skill than those at the elite or expert stage and we refer to this stage of development as “eminence.” This paper explores the notion of eminence and provides converging forms of evidence for the division between expertise and eminence. Moreover, it explores the implications of this division for the further examination of skill acquisition across the lifespan.

The expansion of the field of sport expertise over the past 20 years has been remarkable. As a result, our understanding of the varying factors affecting athlete development and motor skill acquisition has developed significantly (see Baker and Farrow, 2015, for a review). For instance, the need for an extensive period of *deliberate practice* (Ericsson et al., 1993) is now established as a basic requirement for the development of expertise, but the concept of deliberate practice is relatively new to the science of human skill acquisition and expertise. Similarly, the current focus on psychological qualities such as grit (Duckworth et al., 2007) and self-regulation (McCardle et al., 2019), perceptual cognitive issues such as the quiet eye (Vickers, 2016) and representative learning designs (Pinder et al., 2011), and environmental factors such as quality of the early developmental environment (Baker et al., 2009) and the role of athletic parents (Wilson et al., 2019) each reflect relatively new developments in this field of research.

Recently, there has been a push toward more sophisticated research designs to continue the advancement of our understanding of sport expertise. For example, there has been an increased focus on longitudinal research (e.g., Elferink-Gemser et al., 2007; Huijgen et al., 2010; Till et al., 2015), an important improvement over evidence from correlational and qualitative studies. Despite these improvements in study design, Baker et al. (2015) suggested that our understanding of the process of athlete development is limited due to variations in terms used to categorize skill groups (cf. Swann et al., 2014). They proposed a comprehensive taxonomy for categorizing skill levels from beginning stages to most advanced levels. Having clearer delineations between skill levels is important for understanding differences between groups and improving study designs to explore the mechanisms of these differences. Moreover, they (and others) suggested peak levels of performance could be delineated beyond the “expert” level.

Even in a population of performers at the highest levels of performance and competition (e.g., participants in professional sports or those who compete at Olympic Games), there are those with obvious superiority compared to others in the cohort. For example, Collins et al. (2016) highlighted the differences between those with *multiple* Olympic medals or international “caps” (superchamps) and those with *single* medals wins (champs) and those players who had achieved well at youth levels but did not achieve the same high standard in adulthood. Similarly, Hardy et al. (2017) recently examined differences between athletes who had won multiple medals at the Olympics and World Championships (super-elites) and those who attended these events but did not win a medal (elites; see also Barth et al., 2018). This paper builds a case that athletes who reach this level of achievement possess a more advanced level of skill than those at the elite or expert stage and we refer to this stage of development as “eminence” (see Baker et al., 2015). The Oxford English Dictionary defines eminence as “acknowledged superiority within a particular sphere” which seems an appropriate description for this concept. This paper explores the notion of eminence in North American professional sports, a unique sporting context where success can be measured using a range of performance, attainment, and fame-related variables. With this in mind, we provide several converging forms of evidence for eminence in this population. While the world of professional sport is distinct from other forms of sport, we also explore the implications of this division for the further examination of skill acquisition in sport generally.

## Indicator 1: Most Valuable Player Awards

Among the population of competitors who play professional sports and arguably represent the highest levels of performance in these sports, there is clear distinction between the top players and those at “average” levels of performance. For instance, each year professional sports choose “most valuable player” (MVP) award winners (e.g., the European Golden Shoe in European professional soccer and the Hart Memorial Trophy in the National Hockey League). In this section, we use readily available public access data to describe the proportion of athletes who obtain these achievements in the four major professional sports in North America; the National Basketball Association (NBA), the National Hockey League (NHL), the National Football League (NFL), and Major League Baseball (MLB)^1^.

As the name suggests, MVP awards are given to the player who has made the greatest overall impact in a game, series, or season. Similarly, many leagues have position-specific awards (e.g., in the NHL the Vezina trophy is awarded to the top goaltender and James Norris trophy is awarded to the top defense player). The seasonal awards are typically the result of some system of voting. For instance, the Hart Memorial Trophy in the NHL is given to the player voted most deserving by the Professional Hockey Writers Association. Similarly, the MVP award for MLB is voted on by the Baseball Writers Association of America, and in the NBA, the Maurice Podoloff Trophy is currently assigned based on votes from sportswriters and broadcasters, although until 1980 it was determined by NBA players. There are several sportswriter associations voting on MVP awards in the NFL (e.g., the Professional Football Writers Association, Newspaper Enterprise Association, and Sporting News) although the most notable is arguably the Associated Press Player of the Year Award.

The proportion of athletes who obtained an MVP award depends on the league and the current size of the population of players in that league. In the NHL, for example, there were 793 active players in the 2016–17 season of which only one will be named MVP (odds 1 in 793). Odds in the NBA, MLB, and NFL for seasons starting in 2016 ranged from 1 in 388 in the NBA and 1 in 750 in MLB^2^ to 1 in 1,696 in the NFL.^3^ Obviously, the odds of obtaining position-specific awards are better (e.g., simple odds of winning the Vezina trophy for goaltending in the NHL are 1 in 31) but these values highlight the division between the minority who win major awards and the majority who do not.

### Caveat of the “MVP” Indicator for Eminence

While it would seem that the selection of MVP in a given season would be objective and uncontroversial, there is some evidence that this is not always the case. For example, in 1997, during the period when Michael Jordan was considered by many to be the most dominant player in the NBA, he did not win the MVP despite having led the Chicago Bulls to 69 wins that season, tied for the second most of all-time at that point. The award went to Karl Malone in (arguably) one of the most controversial selections in NBA history. Many felt that because Jordan had won the award on four previous occasions, it was time to give it to someone else (Schoenfield, n.d.). Nevertheless, this MVP selection bias does not appear to be a regular occurrence as there is a discernible standout performer in most seasons. This example also highlights the complex nature of MVP awards and recurring debates that surround them. Namely, it is reasonable to debate whether or not Karl Malone was indeed *more valuable* to his team than Michael Jordan (often such debates focus on the strength of a prospective MVP’s teammates). Regardless of whether a “bias” existed in awarding Karl Malone the MVP, Karl Malone is still objectively one of the greatest players in NBA history (indeed, he is a two-time MVP winner). As such, even when controversy exists in the awarding of MVP awards, the debate seems to be *between* eminent athletes.

## Indicator 2: The Hall of Fame

Sports “Halls of Fame” serve to honor athletes (and others such as managers, writers, etc.) who have made lasting contributions to a sport. In the major professional sports in North America explored above, the eligibility criteria for players to be “elected” to the Hall of Fame vary but involve (1) a minimum length of time since retirement (e.g., 3 years for the NHL and NBA, 5 years for living players from MLB and NFL, although MLB limits this to 6 months if the player is deceased) and (2) election by a selection committee normally made up of media members and/or previous Hall of Fame inductees. Our data on the NBA, NHL, NFL, and MLB indicate a considerably smaller proportion of professional athletes obtain this level of achievement (see Table 1). To illustrate, in MLB, for example, less than 2% of players who have ever played in the league have attained Hall of Fame status. This is echoed in the other professional sports, where the proportion of the overall population that gains Hall of Fame status ranges from approximately 1–4%.

**Table 1:** Proportion of professional athletes who make their respective sports’ Hall of Fame.

Sport	Total *N*^a^	Hall of Fame *N* (%)^b^
Ice Hockey (NHL)	7,596	281 (3.7)
Baseball (MLB)	19,429	261 (1.3)
American Football (NFL)	25,791	279 (1.1)
Basketball (NBA)	4,668	124 (2.7)
Total	57,484	945 (1.6)

*Data from official archives for each professional sport (nhl.com; mlb.com; nfl.com; nba.com)*.

a *Total number of athletes who played in the respective sports up to the 2018–2019 seasons*.

b *Total number of athletes who were inducted into the respective sports Halls of Fame up to the 2018–2019 eligibility periods*.

### Caveat of the “Hall of Fame” Indicator for Eminence

To many, election to the Hall of Fame reflects the ultimate recognition of sustained achievement in these sports. However, this indicator is open to bias from the subjective political and moral opinions of those making the election decisions. For example, MLB has maintained a “permanently ineligible” list since 1920 that includes players generally found to have conspired to influence the outcomes of games for their own benefit. Perhaps the best known of these is Pete Rose, undoubtedly one of the greatest baseball players of all time (e.g., winner of three World Series, 17 All-Star Game appearances in five different positions and two “Golden Glove” awards). To date, Rose has been deemed ineligible due to evidence he bet on baseball games for teams he was playing for or managing. There are also some noteworthy players (e.g., Barry Bonds and Roger Clemens) with exceptional performance statistics suspected of using performance enhancing drugs that have to-date been passed over in Hall of Fame voting. All this to say, there is a level of subjectivity to whom eventually makes it to the Hall of Fame in some sports.

## Indicator 3: Career Length

Successful athletes need to continually adapt to changes in the competition environment and manage age- or injury-related declines that might negatively impact their performance. The high performance sport environment is the ultimate Darwinian “survival of the fittest” system and any athlete who is unable to adapt to these changes is (often rapidly) removed from the system. For example, athletes who relied more heavily on physical performance advantages (e.g., speed and strength) early in their career may have to adjust their game later in their career, perhaps learning new skills or relying more heavily on acquired perceptual-cognitive skills.

Arguably, those who are capable of staying in the environment longer (i.e., longer than average) represent a different cohort than the average performers in this population. Below, by way of example, we provide data for all players from the four major professional sports in North America drafted between 1980 and 1989. These dates were chosen because all of these players had completed their playing careers (see Baker et al., 2013, for more on these data). Table 2 presents the average and standard deviations for career lengths among these groups. These data indicate the average career of a professional athlete in these sports during that decade generally ranged between 5.5 years for athletes playing in the NFL and 8.2 years for players in the NBA. However, the standard deviations suggest considerable range around these mean values.

**Table 2:** Mean career length for populations of athletes in the NBA, NFL, NHL and MLB.

Career length	NBA	NFL	NHL	MLB
**Total**				
Mean	8.2	5.5	7.8	7.3
SD	5.4	4.1	5.9	5.3
**Bottom 95%**				
Mean	7.4	5.0	7.9	6.7
SD	5.0	3.5	5.1	4.6
**Top 5%**				
Mean	17.9	15.2	20.5	19.5
SD	1.5	2.2	1.5	1.9

*The top 5% of players roughly equates to those whose careers were two standard deviations above the mean career length. Data from Baker et al. (2013)*.

**Figure 1:**
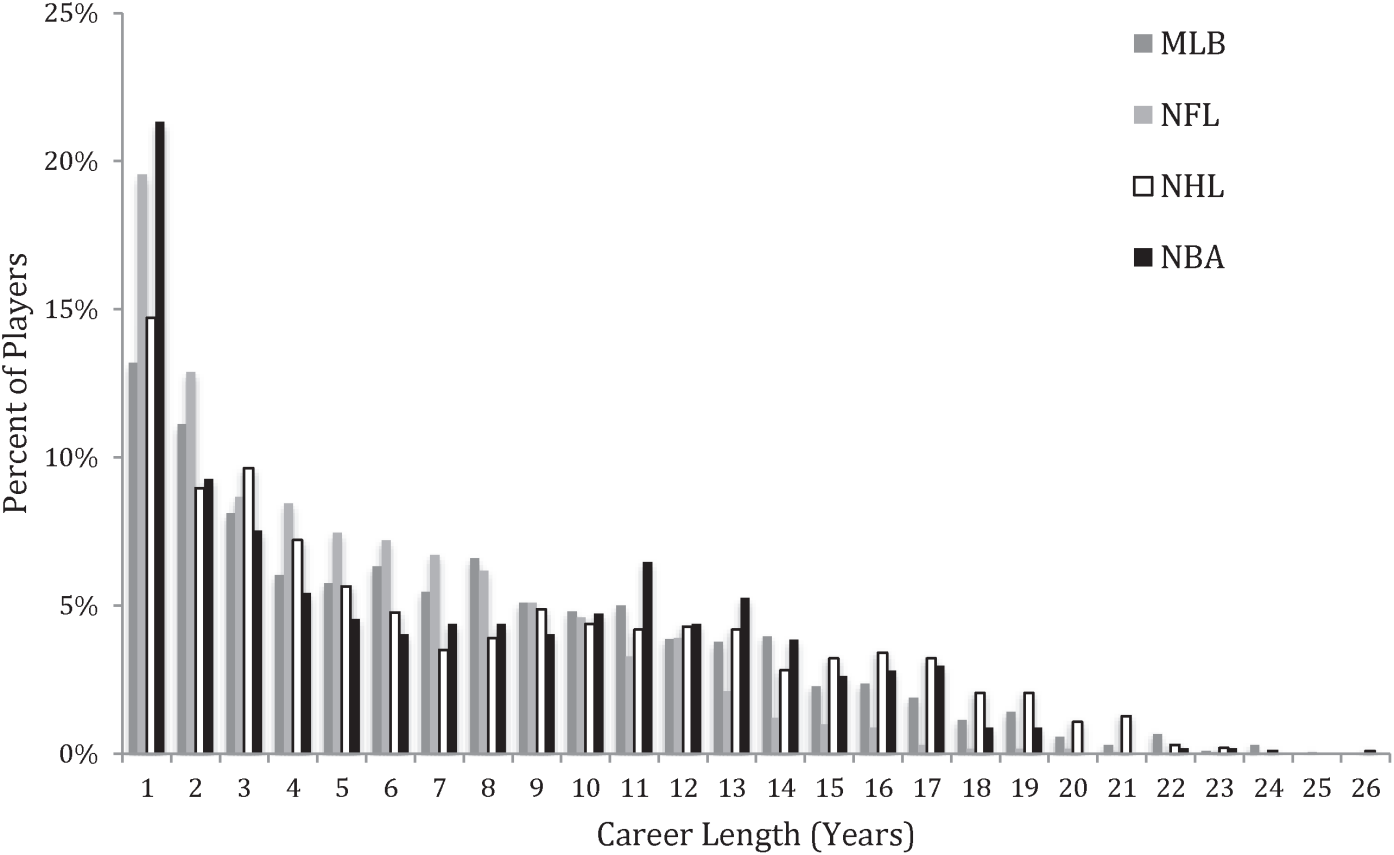
Distribution of career lengths in professional athletes from the NFL, NHL, NBA, and MLB (data current as of end of 2018 seasons).

Preferably, players (and teams) want to have as long a career as possible. However, career length in professional sports is highly negatively skewed (see Figure 1). If we consider the top 5% of players in these leagues (i.e., those whose career length is two standard deviations from the population mean), we see much longer careers in this group. In MLB, the top 5% of players have an average career length of 19.5 (SD = 1.9); similarly, the top 5% of career lengths in the NBA, NHL, and NFL are 18.0 (SD = 1.4) years, 20.5 (SD = 1.5) years, and 15.2 (SD = 2.2) years, respectively.

### Caveat of the “Career Length” Indicator for Eminence

While longer career length indicates important characteristics such as amenability and resiliency for the athlete, this may also mean taking on a different role. More specifically, this indicator of eminence may be confounded by players who have retained a roster spot longer than expected for their ability to maintain team chemistry and morale, for example, rather than their play itself (these players often referred to as “glue guys” or “role players”). Similarly, players who have performed well in prior seasons or who were “marquee” players early in their careers may have longer careers due to “sunk costs” (see Staw and Hoang, 1995). Consequently, considering there is such a small sample of “eminent” players, there is value in triangulating indicators of eminence or considering minute thresholds in order to avoid a distorted understanding.

## Indicator 4: The Lotka-Price Assumption

Another reason for distinguishing a layer beyond “expert” is based in data supporting the Lotka-Price Curve, reflecting a well-known phenomenon about how advancements in various fields occur. For example, examinations of achievement in science have typically supported the conclusion that a relatively small proportion of elites drive progress in a field (e.g., Dennis, 1954; Cole and Cole, 1972; Huber, 1999; Simonton, 1999). Lotka (1926) and Price (1963) hypothesized that scientific progress follows an inverse square law, proposing that the number of scientists publishing *n* papers is proportional to 1/*n*2. This inverse square relationship suggests that for every 100 authors producing a single paper, 25 will produce two papers, 11 with three, and so forth. The Lotka-Price Law also indicates that approximately 50% of the papers published during a given period will be produced by 10% of the actively publishing scientists. In the field of psychology and sport psychology, Simonton (2002) and Baker et al. (2003), respectively, have provided support for the Lotka-Price curve, and this relationship has also been found in a range of performance domains (see Murray, 2003).

**Figure 2:**
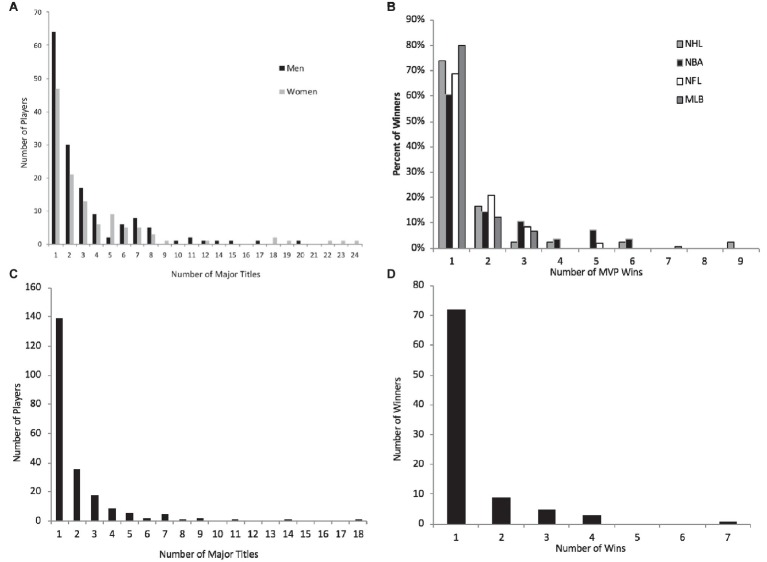
Lotka-Price Curve as demonstrated by **(A)** frequency of wins among players who have won grand slam events in tennis, **(B)** frequency of MVP awards in the NHL, NBA, NFL, and MLB (in %) **(C)** frequency of wins among golfers who have won “major” events on the PGA, and **(D)** frequency of wins among winners of the Boston Marathon (in %).

There is also evidence of this relationship across a range of sport contexts (see for example Petersen et al., 2008; Den Hartigh et al., 2016, 2018). Figure 2 below describes the frequency of wins among players who have won grand slam events in tennis, MVP awards in the NHL, NBA, NFL, and MLB, Professional Golfers’ Association (PGA) golfers who have won “major” events, and winners of the Boston Marathon. In each of these cases, performance and achievement follows the Lotka-Price curve (see also Murray, 2003).

### Caveat of the “Lotka-Price Assumption” Indicator for Eminence

The Lotka-Price profile reflects a relationship between number of athletes in a population and the proportion of those athletes making major achievements. Determining which athletes were among those making “major achievements” in their field could be somewhat arbitrary since achievement in sport varies (e.g., specific positions will have different objectives c.f. goaltenders versus centre-forwards). Further, using the Lotka-Price criterion as an indicator of eminence requires determining a threshold for categorization (i.e., at what point on the curve does the performance become “eminent”?). The caveats to our eminence indicators listed above highlight the complexity of such categorization.

## Need for Multiple Indicators

As noted several times above, it would be valuable to establish a set of clear criteria for the identification of an “eminent” athlete in order to overcome some of the limitations of previous work on exceptional performers. Ideally, given the limitations noted for each individual indicator, this would involve some combination of indicators such as those identified above as well as others yet to be identified. This would have the advantage of triangulating data sources to ensure that any designation is based on multiple indicators thereby providing some evidence of convergent validity. In our list of indicators, there are those that are clearly more objective and grounded in performance (i.e., the Lotka-Price and career length indicators) as well as indicators that may be more subjective and open to social influence (i.e., MVP and Hall of Fame indicators). Using multiple indicators would help reduce the likelihood of any single indicator biasing an athlete’s classification. Moreover, having clear, well thought out indicators would reduce the possibility of conflating “fame” with “eminence,” the former reflecting a social construction (society signifies who becomes famous) and the latter reflecting, at least in our view, an outcome based on robust performance indicators.

It is important to emphasize that the indicators of eminence discussed above are particularly relevant for these professional sports and, undoubtedly, the indicators will need to be adjusted to the sport and context under examination. For example, in European football, Hall of Fame or MVP is not appropriate; here, appearances in international tournaments, matches or goals as well as Ballon D’Or might be better indicators of eminence. However, it is important to note that this could be difficult to determine statistically for each individual performer due to differences in the time course of an athlete’s development. For instance, many athletes in the career length dataset utilized above have not been retired long enough to make it to the Hall of Fame and so the relationship between these indicators in contemporary samples is unknown. Interestingly, every NBA player who has won the MVP award and is eligible for the Hall of Fame has been inducted. It is probable that those at the highest levels of performance as reflected in the Lotka-Price curve will also be those with the longest careers and more likely to achieve Hall of Fame honors. We explore these assumptions below.

## Case Study: The National Hockey League

Now that we have established the case for the eminence level of skill, we examine these indicators in a population of athletes from a single sport to determine the level of agreement between indicators. Our intention in this descriptive analysis is to demonstrate one process by which eminent players could be identified using readily available data.

### Description of the Analysis

The NHL has been in existence since 1917 and has grown from the “original six” teams to the current system with 31 teams from across Canada and the United States. Throughout the history of the NHL, there has been considerable change (e.g., teams added, moved, etc.) and therefore historical samples might have limited relevance for current samples. Given that the indicators have different timescales, not all indicators are possible in contemporary samples (e.g., career length is easy to calculate at the end of a player’s career, but Hall of Fame status may not be determined until decades after a career ends). In this descriptive analysis, we began with identifying NHL players who won the Hart Trophy, established in 1923 for the “most valuable player to his team” and determined how many met other indicators of eminence (Table 3). Moreover, we limited our analysis to players who were active in the pre-1970 NHL. This cut-point, while seemingly arbitrary, reflects an important phase in the history of the league, after the considerable expansion of the league between 1967 and 1970 and is likely a stronger reflection of the current environment. Subsequently, we determined the proportion of players who were also MVPs at some point in their career and were elected to the Hall of Fame. Of the eligible players since 1970 (*n* = 17) who have won the Hart Trophy, only one has not been elected to the Hall of Fame (i.e., 94% agreement).

**Table 3:** Summary of eminence indicators in NHL case study sample.

Name	Position	Hall of Fame ^a^	Career length ^b^	Career points	Lotka-price cutoff ^c^
Bobby Orr	Defense	Yes	12	915	Yes
Phil Esposito	Centre	Yes	18	1,590	Yes
Bobby Clarke	Centre	Yes	15	1,210	Yes
Guy Lafleur	Right Wing	Yes	17	1,353	Yes
Bryan Trottier	Centre	Yes	18	1,425	Yes
Wayne Gretzky	Centre	Yes	20	2,857	Yes
Brett Hull	Right Wing	Yes	19	1,391	Yes
Mark Messier	Centre	Yes	25	1887	Yes
Mario Lemieux	Centre	Yes	17	1,723	Yes
Sergei Fedorov	Centre	Yes	20	1,179	Yes
Eric Lindros	Centre	Yes	13	865	No
Dominik Hasek	Goalie	Yes	16	NA ^d^	Yes
Chris Pronger	Defense	Yes	16	698	No
Joe Sakic	Centre	Yes	21	1,641	Yes
Jose Theodore	Goalie	No	18	NA ^d^	Yes
Peter Forsberg	Centre	Yes	13	885	No
Martin St. Louis	Right Wing	Yes	17	1,033	Yes
Average			17.4	1376.8	
SD			3.2	538.0	
% Agreement		94%			82%

a *Player in the Hall of Fame*.

b *Career length in years*.

c *Did player meet Lotka-Price cutoff of 898 career points*.

d *Goalie data not applicable*.

**Figure 3:**
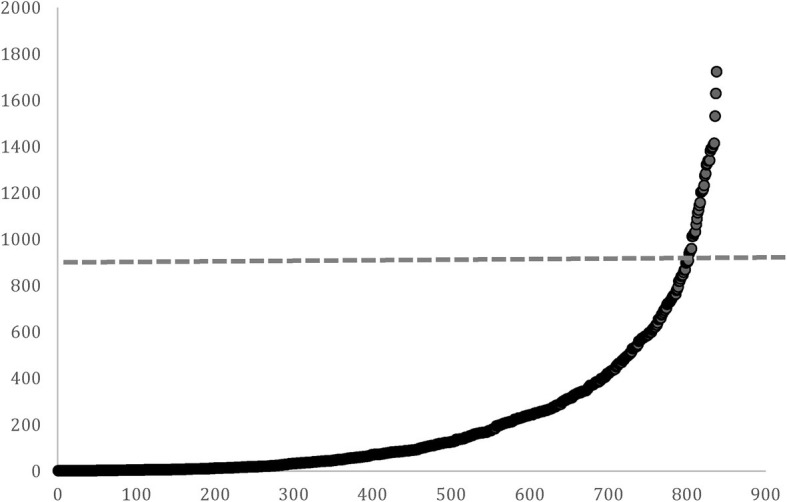
Lotka-Price curve for career points (y-axis) among NHL players drafted between 1980 and 1989 (x-axis). Note: dashed line represents cutoff for top 5% on this outcome.

Next, we considered this same group of 17 players on career length relative to the career lengths determined by Baker et al. (2013). In their analyses of NHL players from 1980 to 1989, they found that the average career length was 7.83 years (SD = 5.84 years). In the MVP sample, career length was 17.4 years (SD = 3.2), suggesting a considerably longer career than the average player. Finally, we plotted the Lotka-Price curve for “career points” (including goals and assists) for all skating players (*N* = 843; goaltenders were eliminated) in the NHL from 1980 to 1989, which was the most contemporary dataset we could use where players were likely to have completed their careers. We then identified a “cutoff” to identify the top 5% of performers on this outcome (i.e., two standard deviations from the mean, 898 career points, see Figure 3). If we compare this to the average career points for our sample of MVPs, their average (*M* = 1376.8) is notably higher than the cutoff used for our Lotka-Price indicator although there is some variability among the players. All but three of the players met the Lotka-Price assumption, reflecting an agreement of 82%. All in all, this exploratory analysis indicates good consistency between indicators and provides some evidence of convergent validity. However, continued attention to validating these indicators would be important (e.g., how do subjective indicators of eminence change over time such as reflected in “best ever” lists of players, etc.).

## The Importance of the Eminence Stage for Understanding Athlete Development

Although this paper argues for a new skill distinction for eminent performers, this view is not without some limitations. First, it assumes these groups can be easily divided into meaningful levels of skill (e.g., intermediate, advanced, expert, and eminent), which runs counter to the notion of skill acquisition as a continuous, on-going process without delineation points reflecting different skill groups. Moreover, it could be easily argued that any population of “eminent” individuals could be further sub-divided into infinitely more detailed categories (e.g., the very eminent).

While these concerns are reasonable, the empirical study of expertise often requires the use of experimental (i.e., between subject) designs that require categorization of individuals into groups for the basis of comparisons. As such, having stricter criteria for accurately grouping individuals is important. Further, study designs in expertise research are still dominated by the expert versus novice/non-expert comparison despite calls for the past 25 years to expand the skill groups being considered (e.g., Abernethy et al., 1993; Baker et al., 2015). Importantly, skill-group classifications need to be justified using multiple indicators. In this paper, we have argued for several indicators relevant for athletes in the major professional sports in North America but the argument holds for other professional and competitive sports, as well as for other levels of skill (i.e., stronger arguments for classifying “novices” or “near elites”).

In the sections above, we delineate a stage above the expert level on the basis that this group might be important for advancing our understanding of exceptional attainment. For example, a dominant paradigm in the field of expertise development over the past 25 years has focused on the role of “deliberate practice” in explaining expert achievement. Those who argue against the role of deliberate practice as a sufficient explanation often evoke the names of eminent individuals (e.g., Amadeus Mozart, William Shakespeare, see Feldman and Katzir, 1998) as evidence against the practice account. Their argument is that (1) these individuals are exceedingly rare in any domain and (2) they make achievements that are qualitatively and quantitatively greater than the “average” high-level performer. On the one hand, this argument might simply reflect the obvious variability within a population of expert performers. That is, individuals who have reached the pinnacle of achievement in a domain (e.g., meeting one or more of the criteria discussed above) represent a different group of performers than “average experts” — similar to how experts are distinguishable from near-experts (i.e., those at a level of performance slightly lower than expert). Furthermore, the existence of eminent performers among expert samples may add important, but obfuscated, variation within studies. For example, whether studies measure performance metrics, accumulated practice, or differences in perceptual cognitive skills, it is not well understood how (or if) eminent athletes may act as statistical outliers that have an influence on aggregate group statistics. Determining the validity of this designation has the potential to promote greater understanding of the highest levels of skill development.

An eminent stage of development could also further our knowledge regarding the influence of different predictors/constraints on the highest levels of achievement. Already, researchers have identified intriguing factors that may differentiate multiple medalists from non-medalists including personality traits such as of selfishness and ruthlessness (see Hardy et al., 2017) and continued examination of this group, and how they differ from “experts” could lead to important insights. It is perhaps important to note that, increasingly, it is this ultimate level of achievement that coaches, athletes, and governments are interested in understanding. For example, the re-structuring of Canada’s high performance sport system was designed to “Own the Podium” during Olympic competition, rather than to simply participate, with an emphasis on obtaining top-3 finishes. Exploring this group would also facilitate superior study designs to prevent skill-level variance from “washing out” any differences between skill groups.

Ultimately, identifying those with the highest levels of attainment in a domain will help us better understand the stages of career development as well as the factors affecting this development. Moreover, it may provide us with insight regarding the long-term consequences of involvement at the most extreme level of performance (e.g., effects of extreme training/diet/weight loss, etc.).

## Concluding Thoughts

The past few years have seen increased emphasis on the need to improve research designs in skill acquisition and expertise research (e.g., Baker et al., 2015; Johnson et al., 2018, see also Abernethy et al., 1993). In the sections above, we justify a distinction at the highest levels of attainment to capture athletes who have achieved “eminent” status and demonstrate how this distinction might be identified in a population of elite athletes. That said, explorations in this new population will come with their own methodological constraints. For instance, the number of eminent athletes is small to begin with, so studies of this type will almost always be limited in sample size. Even in the case study presented above, there were only 17 eminent players in a 34-year period (all players awarded the Hart Trophy after 2005 are still active in the league), which prevents the use of more complex statistical approaches. While it may be attractive to relax the categorizations used to identify the eminent group, it is likely more important to keep the definition strict. Having stronger criteria for identifying and classifying athletes into skill groups will ultimately assist researchers seeking to understand the factors facilitating and impeding long-term development, as well as the mechanisms underpinning truly exceptional sporting achievement.

## Data Availability

The raw data supporting the conclusions of this manuscript will be made available by the authors, without undue reservation, to any qualified researcher.

## Ethics Statement

All data presented in this article are from secondary, publicly available databases and institutional ethics approval for this analysis was not required as per applicable institutional and national guidelines and regulations.

## Author Contributions

JB was the primary author and wrote the manuscript with the help and guidance of co-authors. JS contributed to formulate the structure of this project, helped develop the rationale, and edited the manuscript. SL also helped develop the rationale, and edited the manuscript. NW contributed to the development of the manuscript with JB, and co-wrote and edited the manuscript.

### Conflict of Interest Statement

The authors declare that the research was conducted in the absence of any commercial or financial relationships that could be construed as a potential conflict of interest.

## Footnotes

^1^Data were drawn from hockeydb.com, basketball-reference.com, baseball-reference.com, and pro-football-reference.com. Note: All data presented in this article are from secondary, publicly available databases and institutional ethics approval was not required.

^2^In MLB, there is a 25 player “active roster” and a 40 player “expanded roster” that includes all players in the development system eligible for play throughout the season. Although the expanded roster is technically eligible for the MVP award, we have used the smaller active roster in our calculations.

^3^Active players were determined by multiplying allowable roster size by number of teams. All data from Wikipedia.org.
